# The crystal structure of *Leishmania major N*^5^,*N*^10^-methylenetetrahydrofolate dehydrogenase/cyclohydrolase and assessment of a potential drug target^☆^

**DOI:** 10.1016/j.molbiopara.2011.11.004

**Published:** 2012-02

**Authors:** Thomas C. Eadsforth, Scott Cameron, William N. Hunter

**Affiliations:** Division of Biological Chemistry and Drug Discovery, College of Life Sciences, University of Dundee, Dow Street, Dundee DD1 5EH, UK

**Keywords:** Antifolate, Cyclohydrolase, Dehydrogenase, Drug target, Leishmania, Trypanosoma

## Abstract

Three enzyme activities in the protozoan *Leishmania major*, namely *N*^5^,*N*^10^-methylenetetrahydrofolate dehydrogenase/*N*^5^,*N*^10^-methenyltetrahydrofolate cyclohydrolase (DHCH) and *N*^10^-formyltetrahydrofolate ligase (FTL) produce the essential intermediate *N*^10^-formyltetrahydrofolate. Although trypanosomatids possess at least one functional DHCH, the same is not true for FTL, which is absent in *Trypanosoma brucei*. Here, we present the 2.7 Å resolution crystal structure of the bifunctional apo-DHCH from *L. major*, which is a potential drug target. Sequence alignments show that the cytosolic enzymes found in trypanosomatids share a high level of identity of approximately 60%. Additionally, residues that interact and participate in catalysis in the human homologue are conserved amongst trypanosomatid sequences and this may complicate attempts to derive potent, parasite specific DHCH inhibitors.

## Graphical abstract

**Graphical abstract:**

The crystal structure of *Leishmania major N*^5^,*N*^10^-methylenetetrahydrofolate dehydrogenase/*N*^5^,*N*^10^-methenyltetrahydrofolate cyclohydrolase is used to assess the potential of this bifunctional enzyme as a drug target.

**Figure:**
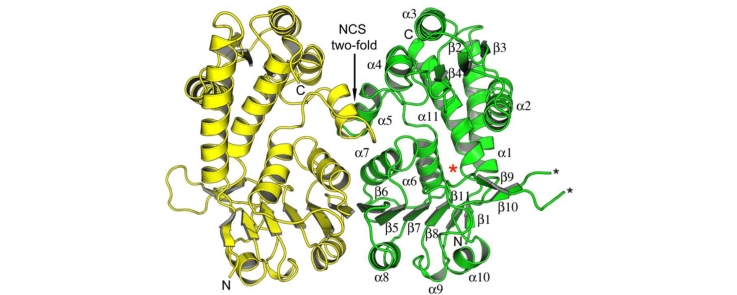

**Highlights:**

► We report the structure of *Leishmania major* methylenetetrahydrofolate dehydrogenase/cyclohydrolase. ► Sequence–structure comparisons are carried out with homologues from kinetoplastids and the human host. ► The potential of this bifunctional enzyme as a drug target is assessed. ► The similarities between parasite and human enzymes suggest a difficult target for drug discovery.

## 1 Introduction

The kinetoplastid protozoan *Leishmania* and *Trypanosoma* sp. are the causal agents for a range of serious parasitic infections [1]. Although drugs are available for the treatment of these diseases they are toxic, costly and with low efficacy [2]. Increasing levels of drug resistant parasites [3] further complicate this biomedical problem and the need for improved diagnostic methods and treatments is as great now as ever. Modern genomic-driven research is helping to unravel the molecular and cell biology of these primitive eukaryotes; for example a large dataset is available on which genes might encode essential activities [4]. We also have an improved understanding of what types of molecules are likely to provide drug targets or appropriate lead compounds and it is timely to identify and critically assess potential targets that might underpin future efforts in drug discovery [5]. With this goal in mind we identified the bifunctional *N*^5^,*N*^10^-methylenetetrahydrofolate dehydrogenase/cyclohydrolase, an enzyme involved in folate metabolism, as a potential drug target in *Leishmania*.

**Fig. 1:**
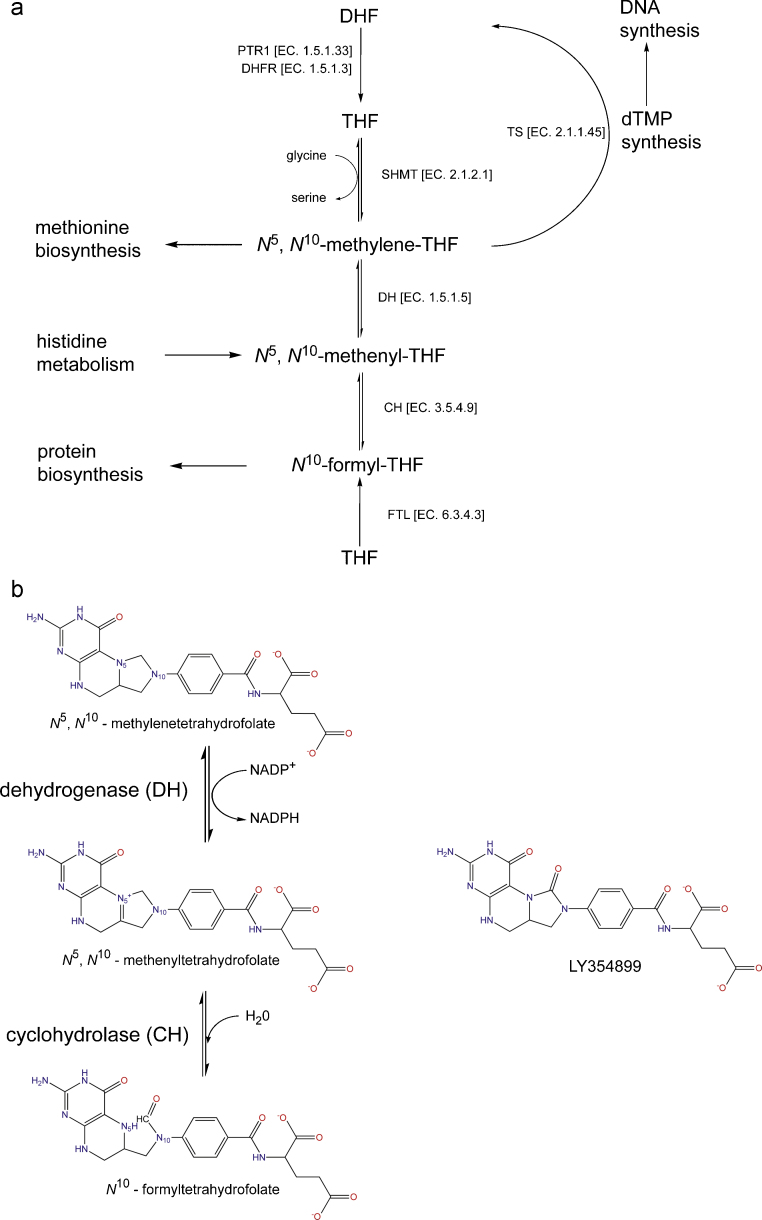
An overview of the folate metabolism pathway in formation of essential intermediates in *Leishmania*. (a) Dihydrofolate (DHF) is converted to tetrahydrofolate (THF) by the actions of dihydrofolate reductase (DHFR) and/or pteridine reductase (PTR1). THF is modified by serine hydroxymethyl transferase (SHMT) or formyltetrahydrofolate ligase (FTL) and the interplay between three substituted intermediates of THF is controlled by the bifunctional dehydrogenase cyclohydrolase (DHCH). Intermediates generated at each stage are utilized by other pathways, for example *N*^5^,*N*^10^-methylenetetrahydrofolate is used by thymidylate synthase (TS). (b) The action of DHCH maintains the required levels of methenyl-, methylene-, formyl-tetrahydrofolate molecules, first by an NADP^+^ dependent oxidation from *N*^5^,*N*^10^-methylenetetrahydrofolate to form *N*^5^,*N*^10^-methenyltetrahydrofolate, then to *N*^10^-formyltetrahydrofolate by the cyclohydrolase activity. The inhibitor LY354899 is also shown. Figure produced using ChemDraw.

Folate and derivatives are essential cofactors in the biosynthesis of thymidine, purines, glycine, methionine, initiator fMet-tRNA and also in the metabolism of histidine and serine (Fig. 1a) [6]. It is not surprising that enzymes involved in folate-dependent pathways, e.g. dihydrofolate reductase (DHFR), are important antimicrobial and anticancer drug targets [7,8]. Trypanosomatids are auxotrophic for folates and pterins [9] and reliant on uptake and salvage mechanisms to maintain the required level of these important compounds. Inhibition of DHFR should, in principle, provide a route to treat trypanosomatid infections. However, the presence of a pteridine reductase (PTR1) able to reduce dihydrofolate (DHF) to tetrahydrofolate (THF), i.e. catalyze the same reaction as DHFR, helps to compromise the use of such inhibitors [10]. Promising PTR1 inhibitors have been identified [11–15] in support of a strategy to develop a combination treatment with known DHFR inhibitors to cut-off the supply of reduced pterins/folates. The use of drug combinations might also serve to alleviate the development of drug resistance [13,15]. Here, we turn our attention onto enzymes that maintain the required levels of *N*^10^-formyltetrahydrofolate, a key intermediate supporting protein synthesis.

THF is converted to *N*^5^,*N*^10^-methylenetetrahydrofolate by serine hydroxymethyl transferase or the glycine cleavage system [6]. A two-step reaction follows, initially an NADP^+^ or NAD^+^ dependant oxidization of *N*^5^,*N*^10^-methylenetetrahydrofolate to the intermediate *N*^5^,*N*^10^-methenyltetrahydrofolate catalyzed by *N*^5^,*N*^10^-methylenetetrahydrofolate dehydrogenase (DH) and the methenyl derivative subsequently hydrolyzed to *N*^10^-formyltetrahydrofolate by *N*^5^,*N*^10^-methenyltetrahydrofolate cyclohydrolase (CH, Fig. 1b) [16]. *N*^10^-formyltetrahydrofolate also results from addition of formate onto THF in a reaction catalyzed by formyltetrahydrofolate ligase (FTL) [17]. Humans possess a cytosolic trifunctional C-1-tetrahydrofolate synthase with DHCH and FTL activities, in addition to a mitochondrial bifunctional DHCH and monofunctional FTL [18]. Structures of cytosolic human DHCH (*Hs*DHCH) [19,20] have formed the basis of some mechanistic understanding [21].

All trypanosomatids possess this dehydrogenase and cyclohydrolase activity with a cytosolic bifunctional enzyme (DHCH). In *Leishmania major* and *Trypanosoma cruzi* a separate enzyme catalyzes the FTL reaction but this enzyme is absent in *Trypanosoma brucei* [22,23]. The cytosolic DHCH activity appears essential in *L. major* since knockouts of the disomic copies were not possible without ectopic expression of FTL to provide an alternative route to *N*^10^-formyltetrahydrofolate [24]. Since trypanosomatids are purine auxotrophs [25] then the essential requirement for a supply of *N*^10^-formyltetrahydrofolate is likely due to its contribution to production of fMet-tRNA and ultimately for protein synthesis. Several *Leishmania* species, including *Leishmania donovani*, possess an additional putative mitochondrial enzyme (DHCH2) that shares between 27 and 31% sequence identity with DHCH. Although DHCH2 is larger that the cytosolic enzyme, the residues predicted to interact with the substrate are conserved across all species [23]. In *L. major* a pseudogene, *dhch2*, is noted [Genedb, http://www.genedb.org]. This implies that *N*^10^-formyltetrahydrofolate is synthesized in the cytosol and then transported into the mitochondria for use in fmet-tRNA [23]. The presence of DHCH activity in the mitochondria of some trypanosomatids is a complicating factor. Its presence could compromise inhibition of the cytosolic enzyme if *N*^10^-formyltetrahydrofolate can be transported out of the organelle to the cytosol. Inhibition of both DHCH and DHCH2 might then be necessary to affect the parasite. In *L. major*, lacking a functional DHCH2, FTL activity might provide a by-pass of DHCH inhibition and that would have to be taken into consideration.

We report the crystal structure of *L. major N*^5^,*N*^10^-methylenetetrahydrofolate dehydrogenase/cyclohydrolase (*Lm*DHCH), detailed structure–sequence comparisons with trypanosomatid orthologues, with the human orthologue and explore the potential for the development of potent trypanosomatid specific inhibitors against this essential enzyme.

## 2 Methods

### 2.1 Cloning

The *dhch1* gene, encoding *Lm*DHCH, was identified in Genedb (http://www.genedb.org, accession number LmjF26.0320). Genomic DNA from *L. major* (Friedlin strain) was the template for PCR with the following primers designed to amplify the open reading frame with NdeI and BamHI restriction sites (bold), respectively: 5′-**CAT-ATG**-CCG-TCT-GCT-CAG-ATC-AT-3′, 5′-**GGA-TCC**-CTA-TGA-TAC-GCC-GAA-GCG-A-3′. The PCR product was ligated into pCR-BluntII-TOPO vector using the Zero Blunt TOPO PCR cloning kit (Invitrogen). The gene was then excised from TOPO with NdeI/BamHI and ligated into a modified pET15b (Novagen) containing a Tobacco Etch Virus (TEV) protease recognition sequence in place of a thrombin recognition sequence (pET15bTEV). This results in recombinant expression of a product carrying an N-terminal hexa-histidine tag (His-tag), which is cleavable with TEV protease. The recombinant plasmid was amplified in XL-1 blue *Escherichia coli*, and the gene sequence verified, before being transformed into *E. coli* BL21 (DE3) (Stratagene) for protein production.

### 2.2 Purification

Cells were cultured in 1 L flasks at 37 °C with shaking (200 rpm) in auto induction media [26] supplemented with 50 mg L^−1^ carbenicillin until an OD_600_ of 0.6 was reached. The temperature was subsequently reduced to 21 °C overnight. Cells were collected by centrifugation (4 °C at 4000 × *g* for 30 min). Cells were resuspended in 20 mL of buffer A (50 mM Tris–HCl, 250 mM NaCl, 20 mM imidazole, pH 7.5) with the addition of DNAse (200 μg) and an EDTA-free protease inhibitor tablet (Roche) prior to two rounds of lysis in a French press pressure cell under 16,000 psi. The resulting homogenate was centrifuged (4 °C at 37,500 × *g* for 30 min) and the supernatant loaded onto a pre-equilibrated and Ni^2+^ charged HisTrap HP 5 mL column (GE Healthcare) with the subsequent application of a linear gradient of 20 mM to 1 M imidazole in buffer A. Samples containing *Lm*DHCH were pooled and His-tagged TEV protease was added at 1 mg TEV per 20 mg protein. The mixture was dialysed using snakeskin tubing (10 kDa MWCO) against buffer A for 4 h at room temperature. The sample was again passed over a HisTrap HP 5 mL column to remove the TEV protease, uncleaved *Lm*DHCH, the cleaved tag and histidine-rich proteins from *E. coli*. The *Lm*DHCH was then concentrated and applied to a Superdex 200 26/60 column (GE Healthcare) pre-equilibrated with buffer A. Analysis of the elution profile showed that the main peak eluted with an approximate molecular mass of 65 kDa, indicating *Lm*DHCH forms a dimer. The buffer was exchanged to 50 mM Tris–HCl, 100 mM NaCl, pH 7.5 and the enzyme concentrated to 9 mg mL^−1^ for crystallization trials. The final *Lm*DHCH preparation was estimated to be greater than 95% pure by SDS-PAGE and MALDI-TOF mass spectrometry, with a yield of protein of approximately 5 mg L^−1^ of cell culture. Protein concentration was calculated using the theoretical extinction coefficient of 6320 M^−1^ cm^−1^ using ProtPram [27].

### 2.3 Crystallization and data collection

Crystallization trials were carried out using a Phoenix Liquid Handling System (Art Robins Instruments/Rigaku) with several commercial screens, including MPD, PEG, Classics and ammonium sulfate (Hampton Research) using a 1:1 ratio of 100 nL of protein solution and an equivalent volume of reservoir solution equilibrated against 70 μL reservoir at 20 °C. Crystals were observed after three days with a reservoir of 20% PEG 3350 and 0.2 M ammonium acetate. Optimization in 2 μL drops using the hanging drop vapor diffusion method gave orthogonal blocks with approximate dimensions 0.15 mm × 0.2 mm × 0.1 mm. Single crystals were transferred to a cryo-solution containing the original reservoir solution supplemented with 40% glycerol prior to flash freezing at −173 °C. Crystals were characterized in-house with a Micromax-007 rotating anode generator and R-AXIS IV^++^ dual image plate detector (Rigaku), prior to storage in liquid nitrogen. X-ray diffraction data were then collected at beam line I04 at the Diamond Light Source. Integration and scaling of data were carried out using MOSFLM [28] and SCALA [29]. The crystal displayed space group *C*222_1_ with unit cell lengths *a* = 117.2 Å, *b* = 220.08 Å and *c* = 56.31 Å. The molecular mass of a subunit is 31.9 kDa, and the asymmetric unit consists of two subunits giving a *V*_M_ of 2.8 Å^3^ Da^−1^ and solvent content of approximately 55%.

### 2.4 Structure determination

The structure was solved by molecular replacement and refined to 2.7 Å resolution. The search model was a monomer of the *E. coli* enzyme (*Ec*DHCH) which shares 46% sequence identity, (Protein Data Bank (PDB) code 1B0A) [30] with all side chains removed using CHAINSAW [31]. Rotation and translation functions were solved with PHASER [32] in the Collaborative Computational Suite 4 [33]. The positions of two molecules, forming a dimer were identified with a log likelihood gain of 364. Following rigid-body refinement in REFMAC5 [34], the side chains were built into electron and difference density maps and iterative rounds of restrained refinement carried out with Babinet scaling, electron and difference density map inspection, model manipulation and the addition of solvent molecules using COOT [35] and REFMAC5. Tight non-crystallographic symmetry (NCS) restraints were initially applied and subsequently released. In addition, Translation/Libration/Screw analysis (TLS) [36] was applied. Model quality was checked using MolProbity [37] and crystallographic statistics are summarized in Table 1. Structural superpositions were calculated using LSQKAB [38], domain motion calculated using DynDom [39] and active site volumes calculated using the software program ICM PocketFinder (MolSoft Limited). Figures were prepared using PyMOL (DeLano Scientific). Sequence alignments were calculated using ClustalW [40] and visualized using Aline [41].

## 3 Results and discussion

### 3.1 Overall structure and comparisons

**Fig. 2:**
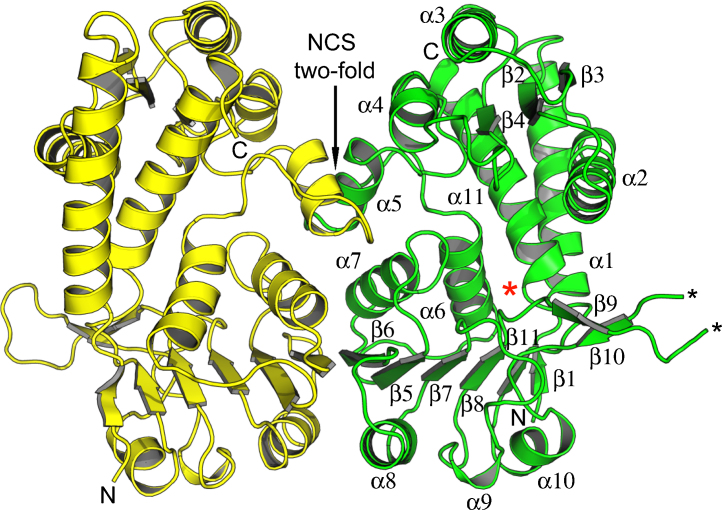
A cartoon representation of the *Lm*DHCH homodimer. Subunit A is yellow, B is green. The elements of secondary structure of subunit B are labeled in addition to the N- and C-termini. A small section of a loop (residues 248–250, highlighted with black asterisks) is absent in subunit B due to disorder.

The crystal structure of *Lm*DHCH has been determined at 2.7 Å resolution. The asymmetric unit is a homodimer and subunits are labeled A and B (Fig. 2). There was no evidence of a monomer in solution and the presence of a stable dimer is consistent with gel filtration data and in common with all other DHCH orthologues [19,42]. The first and last few residues (1–2 and 1–3, 297–298 in chains A and B, respectively) are disordered and an additional three residues are disordered in chain B (248–250). Superposition of subunit A onto subunit B gives an RMSD of 1.7 Å over 286 main chain Cα atoms. This high value reflects differences in domain positions, which will be discussed, and in a loop adjacent to the active site (residues 240–255; RMSD 4.1 Å over 13 Cα). This loop is disordered and absent in other DHCH structures and in *Lm*DHCH, although modelled satisfactorily, elevated thermal parameters are noted.

**Fig. 3:**
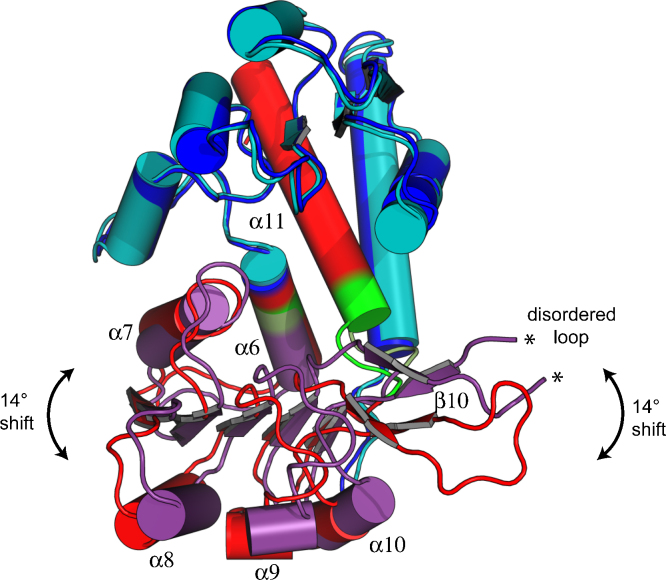
An overlay of the *Lm*DHCH subunits based on superposition of N-terminal domains. The N-terminal domains (chain A – blue, chain B – cyan) agree closely however a pivot (shown in green for chain A and forest for chain B) causes the C-terminal domains (chain A – red, chain B – magenta) to differ by approximately 14°. Such a shift appears accentuated particularly around the loop leading to β10.

The *Lm*DHCH subunit consists of 298 residues displaying a high level of secondary structure; 11 α-helices and 11 β-strands (Fig. 2). The polypeptide folds into N- and C-terminal domains comprising residues 5–147 and 148–298, respectively. A cleft is formed between the two domains and at one end an NADP^+^ binding site, typical of a Rossmann fold (βαβαβ), is formed. The substrate-binding site is at the other end of the cleft. Analysis of the domains of *Lm*DHCH from the crystallographically independent chains shows a significant pivot around a hinge (residues 150–152 and 274–280) with a rotation of almost 14° evident (Fig. 3). The hinge involves α6 and the loop leading into α11. A similar observation has been made in *Hs*DHCH structures [19,20]. The subunits appear in distinct states, with the active site slightly more compressed in one than the other and this may represent a feature of DHCH activity.

**Fig. 4:**
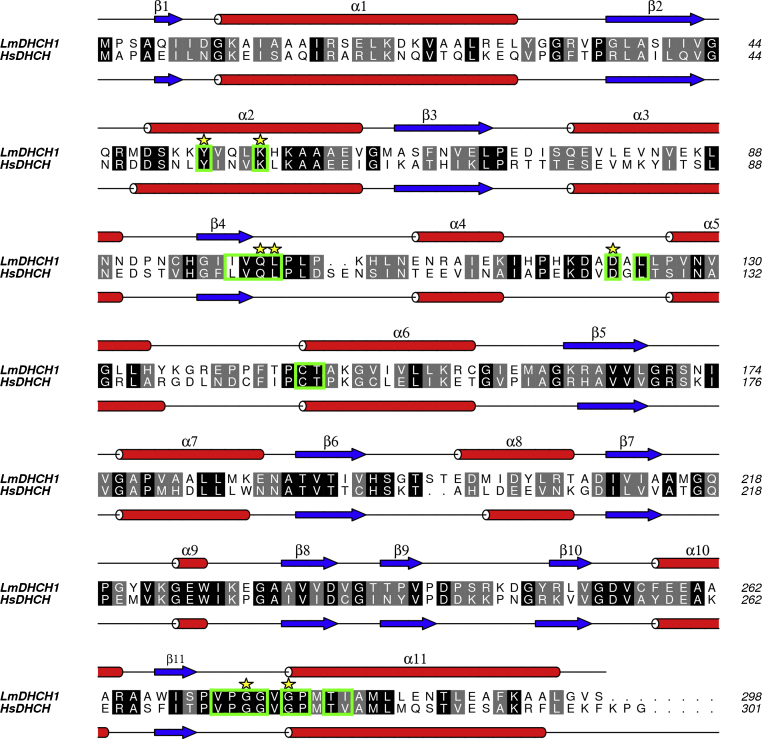
Structure-based sequence alignment of *L. major* and *H. sapiens* cytosolic DHCH enzymes. Helices and strands are red and blue, respectively. Residues that are strictly or highly conserved in *H. sapiens*, *L. major*, *T. brucei*, *T. cruzi* and *L. donovani* enzymes are highlighted in black and grey, respectively. Residues that directly bind ligands (as shown in the *Hs*DHCH structures) are marked with a yellow star and are highly conserved across all species. Residues that are within 5 Å of the bound ligand in the *Hs*DHCH structure are enclosed in green boxes.

**Fig. 5:**
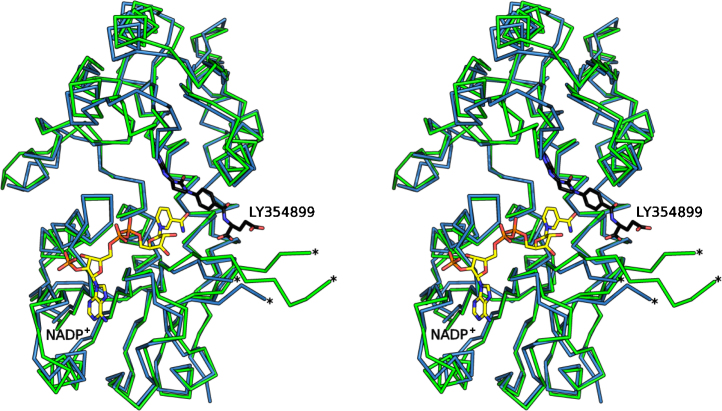
Stereo view Cα trace showing the superposition of a subunit of *Lm*DHCH (green) with an equivalent from *Hs*DHCH (marine) ternary complex with NADP^+^ (yellow) and LY354899 (black) (PDB code 1DIB). In both cases a loop that remains disordered is marked with two asterisks.

The human and *L. major* enzymes share 44% sequence identity (Fig. 4) and the structures are similar. Superposition of *Lm*DHCH subunits onto binary- or tertiary complexes of *Hs*DHCH subunits in the same open or closed forms reveals RMSD values of approximately 1.0 Å over 270 Cα positions (Fig. 5). Superposition of the *Hs*DHCH NADP^+^ binary and an NADP^+^-inhibitor tertiary complexes gives an RMSD of 0.1 Å over 285 Cα atoms, suggesting no gross structural change occurs following inhibitor binding.

### 3.2 The active site

**Fig. 6:**
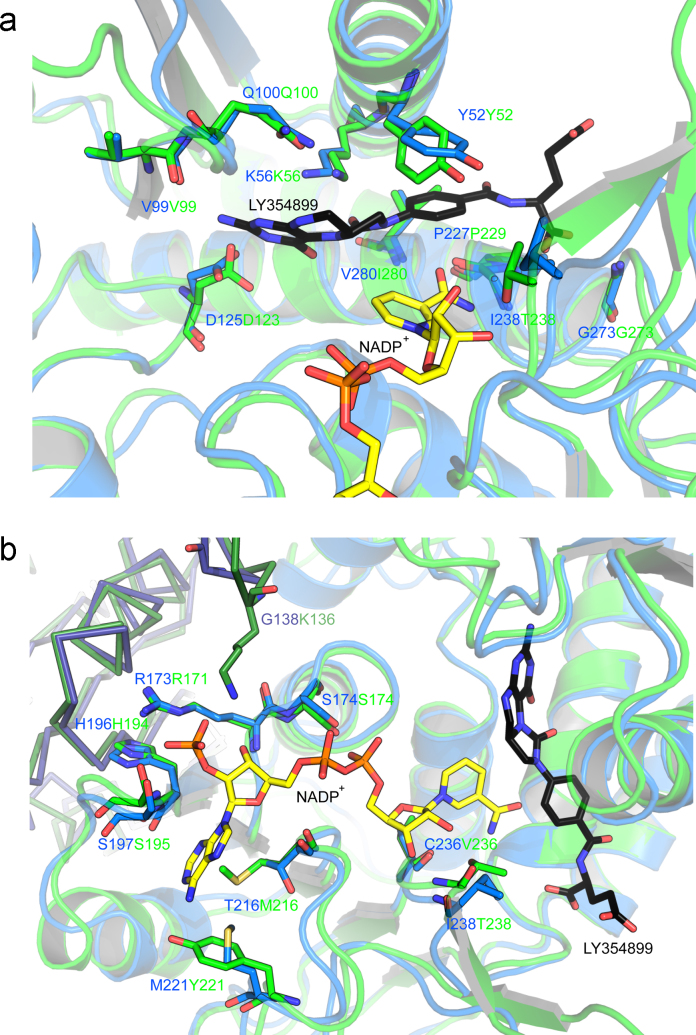
Comparison of the (a) NADP^+^ binding and (b) catalytic sites of *Lm*DHCH (green) with *Hs*DHCH (marine) containing the competitive inhibitor NADP^+^ (yellow) and LY354899 (black), respectively. Residues shown as sticks are within hydrogen bonding distance of the ligands and with labels colored according to species. The side chain of Tyr52 is disordered in the structure of *Lm*DHCH but predicted to be ordered upon ligand binding.

Attempts to obtain structures of *Lm*DHCH ligand complexes proved fruitless and efforts to crystallize *T. brucei* and *T. cruzi* enzymes were also unsuccessful. However the similarities described allow comparisons with *Hs*DHCH complexes (Fig. 5) to inform on aspects of molecular recognition in the active site (Fig. 6) and the potential for developing inhibitors specific for the parasite enzymes over that of the host. Of particular value is the ternary complex of *Hs*DHCH with cofactor and the inhibitor 5,6,7,8-tetrahydro *N*^5^,*N*^10^-caronylfolic acid (LY354899, Fig. 1b, PDB code 1DIB) developed at Lilly Research Laboratories [43]. LY354899 mimics the substrate and inhibits both *Hs*DHCH and *Lm*DHCH with *K*_i_ values of 18 nM and 105 nM, respectively [20,43]. It also inhibits other enzymes involved in folate metabolism, DHFR, thymidylate synthase and glycinamide ribonucleotide formyl transferase [43].

In *Hs*DHCH, 13 residues, mainly using side chains are key in binding NADP^+^ or in creating the cofactor-binding site (Thr148, Arg173, Ser174, Ile176, His196, Ser197, Ala215, Thr216, Gln218, Met221, Cys236, Gly237 and Ile238 [20]). Nine of these residues are strictly conserved in *Lm*DHCH and we note the arginine, serine, histidine combination (Arg171, His194, Ser195) that binds the 2′ phosphate of NADP^+^ is spatially conserved between the two species (Fig. 6a). The four differences involve Thr216 to Met216, Met221 to Tyr221, Cys236 to Val236 and Ile238 to Thr238. The first two provide a hydrophobic surface to bind the adenine moiety. At position 236 it is the main chain that is involved in cofactor binding. The steric effect of changing Ile238 to Thr238 is small, but in *Lm*DHCH the hydroxyl group of Thr238 might form a hydrogen bond to the nicotinamide ribose (data not shown). In addition, Lys156 from the opposing subunit appears primed to interact with the NADP^+^ phosphate in *Lm*DHCH. This is not the case in *Hs*DHCH, as Gly138 occupies the corresponding position. In *Lm*DHCH subunit B a chloride ion, derived from the crystallization conditions, is assigned to a large feature in the electron density maps at a position that mimics the 2′ phosphate binding site (data not shown).

The N-terminal domain primarily forms the catalytic centre. It contains a highly conserved YXXXK motif, starting at Tyr52, found in the small chain dehydrogenase/reductase family, indicative of the substrate-binding site [21]. Seven key residues with respect to substrate binding and catalysis in *Hs*DHCH are Tyr52, Lys56, Gln100, Leu101, Asp125, Gly273 and Gly276 [20,21]. Lys56 acts with Gln100 to provide the right environment to activate substrate and support hydride abstraction by NADP^+^ for the dehydrogenase activity, whilst during the cyclohydrolase reaction it may transfer a proton during formation of *N*^10^-formyltetrahydrofolate. Hydrophobic residues surrounding the catalytic Lys56 (Leu38, Ile40, Ile53, Val55, Leu57, Leu98, Val280 and Leu283), create an environment implicated in maintaining the ɛ-amino group in a non-protonated state, essential for a role in dehydrogenase and cyclohydrolase activity. Tyr52 and Asp125 are important for binding and orienting the substrate for catalysis. The aromatic residue stacks against the *p*-amino benzoic acid moiety of the substrate, the acidic residue binding the pterin head group (Fig. 6b).

In common with the cofactor-binding site there is a high level of conservation at the catalytic centre with all seven key residues strictly conserved. In addition the catalytic Lys56 of *Lm*DHCH is surrounded by aliphatic, hydrophobic side chains (Leu38, Val53, Leu55, Ile98, Ile280 and Leu283) as seen in *Hs*DHCH. Two residues in the vicinity of the catalytic lysine differ between host and pathogen. The positions occupied by Ile40 and Leu57 in *Hs*DHCH are Ser40 and His57 in *Lm*DHCH. These residues are over 6 Å distant from the ɛ-amino group on the other side of the lysine side chain from where substrate will bind (data not shown).

### 3.3 Assessing the potential for structure-based drug design

Several questions should be addressed in assessing a target for potential in antimicrobial drug discovery [5]. Does the target provide an essential function in the pathogen? Is the structure compatible with tight binding of small organic molecules? Is it present only in the pathogen? If an orthologue occurs in the human host, are there sufficient differences to ensure that selective inhibition of the pathogen target can be achieved? We now consider these issues.

On the basis of biological and metabolic considerations DHCH appears a promising drug target in *L. major*. Genetic approaches suggest essentiality in *L. major* [24] and the absence of FTL in *T. brucei* [23] makes DHCH an interesting target in that parasite also as there is no other obvious means by which that organism can produce *N*^10^-formyltetrahydrofolate.

The concept of a druggable protein concerns the properties of a site able to bind molecules with the right physicochemical attributes to make them bioavailable, with high affinity for the target and low toxicity to humans [44]. The optimal target would be a small, well-defined and ordered cavity in the protein, with pronounced hydrophobic components [44]. The active sites of enzymes such as PTR1 and DHFR would be described as ‘druggable’. The volume of their active sites [45] is estimated as 350 Å^3^ and 380 Å^3^, respectively from PDB codes 2X9G and 3CL9. The substrate binding cavity of *Lm*DHCH has a volume of approximately 430 Å^3^ which is a modest increase compared to DHFR and of suitable size to bind drug-like molecules [45]. Indeed drug-like inhibitors of *Hs*DHCH are known supporting this conclusion. These inhibitors were investigated as anticancer agents since the folate pathway produces essential co-factors for cell division [43]. The observation that an *Hs*DHCH inhibitor, LY354899, displays nM inhibition against *Lm*DHCH and an EC_50_ of approximately 1 μM against the parasite itself [24] is supportive of a mode of action being DHCH inhibition with the caveat that there are other potential targets present.

Multiple sequence alignments of *Hs*DHCH with the orthologues from major disease causing members of the *Trypanosomatid* family (*L. major*, *L. donovani*, *T. brucei* and *T. cruzi*) show a relatively low level of overall sequence identity, from 39 to 45%. Comparing just the trypanosomatid sequences the range is 58%, between *L. major* and *T. brucei*, to 96%, between *L. major* and *L. donovani*. The residues involved in substrate/ligand binding and catalysis remain highly conserved across all species (Fig. 4). Such conservation may prove problematic because inhibitors will need to display selectivity for the parasite enzyme over that of the host. This is an issue with the LY354899 inhibitor, which, for reasons that are not obvious, is actually more selective for the human enzyme, *K*_i_ 18 nM as opposed to 105 nM against *Lm*DHCH although we caution that this difference may simply reflect assay variation in different laboratories.

There are two, small, sequence differences in the substrate binding site between host and parasite enzymes; Ile238 and Val280 of *Hs*DHCH corresponding to Thr238 and Ile280 in *Lm*DHCH (Fig. 6b). These residues are not predicted to bind the substrate directly and it would be difficult to attempt to exploit such minor differences in the development of specific inhibitors.

Preventing formation of or destabilizing the *Lm*DHCH dimer may offer a route to inhibition although targeting the interface of oligomeric assemblies to find small molecule drug-like inhibitors presents a significant challenge due to the high level of specificity involved and the relatively large areas involved in oligomerization [46]. Residues on α5, α7, α8 and β6 form the dimer interface of *Lm*DHCH. In particular the β6 strands associate in anti-parallel fashion to create a 12-stranded β-sheet (Fig. 2). The catalytic site is remote from the dimer interface, however, the adenine-end of the cofactor binds nearby indeed Lys136 from one subunit is placed to interact with the 2′-phosphate of the cofactor bound by the partner subunit. However, comparisons indicate a high level of conservation between *Lm*DHCH and *Hs*DHCH in α5, α7 and β6 (Fig. 4) extending to similarities in hydrogen bonding patterns (data not shown).

## 4 Conclusion

The crystal structure of apo-*Lm*DHCH has been determined to 2.7 Å resolution allowing for detailed comparisons with *Hs*DHCH. Our data provide details of the active site and a template for the rational development of selective inhibitors against the enzyme from trypanosomatid parasites. However, two major factors may compromise the search for selective inhibitors. Firstly, the inability to co-crystallize a binary or tertiary complex of any protozoan DHCH has limited efforts to exploit structure-based methods for inhibitor development. Most *Hs*DHCH ligand complexes in the PDB display poorly ordered ligands [20] and DHCH itself may present significant challenges for structure-based approaches to inhibitor development. Secondly, and perhaps most important is the close structural relationship between the host and parasite enzymes not only at the catalytic centre but also at the dimer interface. This probably represents the biggest challenge to early stage drug discovery seeking to develop potent and parasite specific inhibitors of DHCH.

## Figures and Tables

**Table 1:** Crystallographic statistics.

Space group	*C*222_1_
Unit cell lengths *a*, *b*, *c* (Å)	117.22, 220.08, 56.31,
Resolution range (Å)	40.0–2.7 (2.85–2.7)^a^
Wavelength (Å)	0.972
Number of measurements	77501 (11497)
Number of unique reflections	17725 (2631)
Multiplicity	4.4 (4.4)
Completeness (%)	88.4 (90.7)
Mean *I*/*σI*	6.5 (2.8)
Wilson *B* (Å^2^)	60.5
*R*_*merge*_ b	0.134 (0.488)
*R*_*work*_ c	0.231
*R*_*free*_ d	0.290
RMSD bonds (Å)	0.0054
RMSD angles (°)	0.871

Ramachandran analysis (%)	
Favoured	97.8
Allowed	2.0
Outliers	0.2
Protein residues	586
Protein atoms total	4398
Overall *B* (Å^2^) subunit A, B	38.9, 58.4
Waters	22
Overall *B* (Å^2^)	29.3
Cl^−^	1
Overall *B* (Å^2^)	47.2

a Values in parentheses refer to the highest resolution bin of 2.85–2.7 Å.

b *R*_*merge*_ = ∑*h∑i*||(*h*,*i*) − 〈*I*(*h*)〉 ∑*h∑i I*(*h*,*i*).

c *R*_*work*_ = ∑*h* *k* *l*||*F*_*o*_| − |*F*_*c*_||/∑|*F*_*o*_|, where *F*_*o*_ is the observed structure factor and *F*_*c*_ is the calculated structure factor.

d *R*_*free*_ is the same as *R*_work_ except calculated using 5% of the data that are not included in any refinement calculations.
